# Supplementary figures and images for: The phosphatase inhibitor LB-100 creates neoantigens in colon cancer cells through perturbation of mRNA splicing
Source: EMBO Rep. 2024 Apr 10;25(5):10. doi: 10.1038/s44319-024-00128-3 (PMC11094086; doi:10.1038/s44319-024-00128-3)

ATM

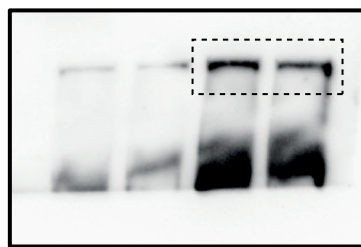

Chk2

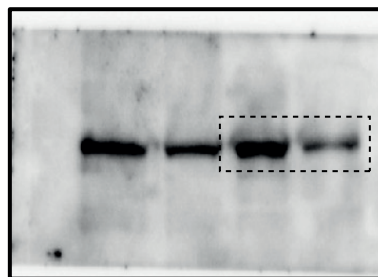

$\alpha$ -Tubulin

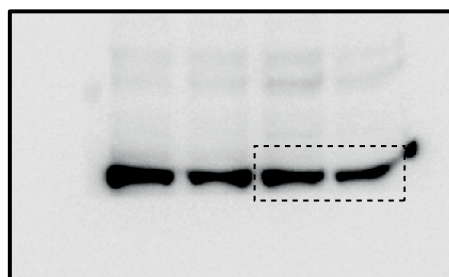

Supplement: Supplementary file 8 — Source data Fig. 3 [file 44319_2024_128_MOESM8_ESM.zip › Source Data Figure 3/Source Data Figure 3.pdf]
