# Supplementary material for: The phosphatase inhibitor LB-100 creates neoantigens in colon cancer cells through perturbation of mRNA splicing
Source: EMBO Rep. 2024 Apr 10;25(5):10. doi: 10.1038/s44319-024-00128-3 (PMC11094086; doi:10.1038/s44319-024-00128-3)
Supplement: Supplementary file 12 — Expanded View Figures [file 44319_2024_128_MOESM12_ESM.pdf]

Expanded View Figures

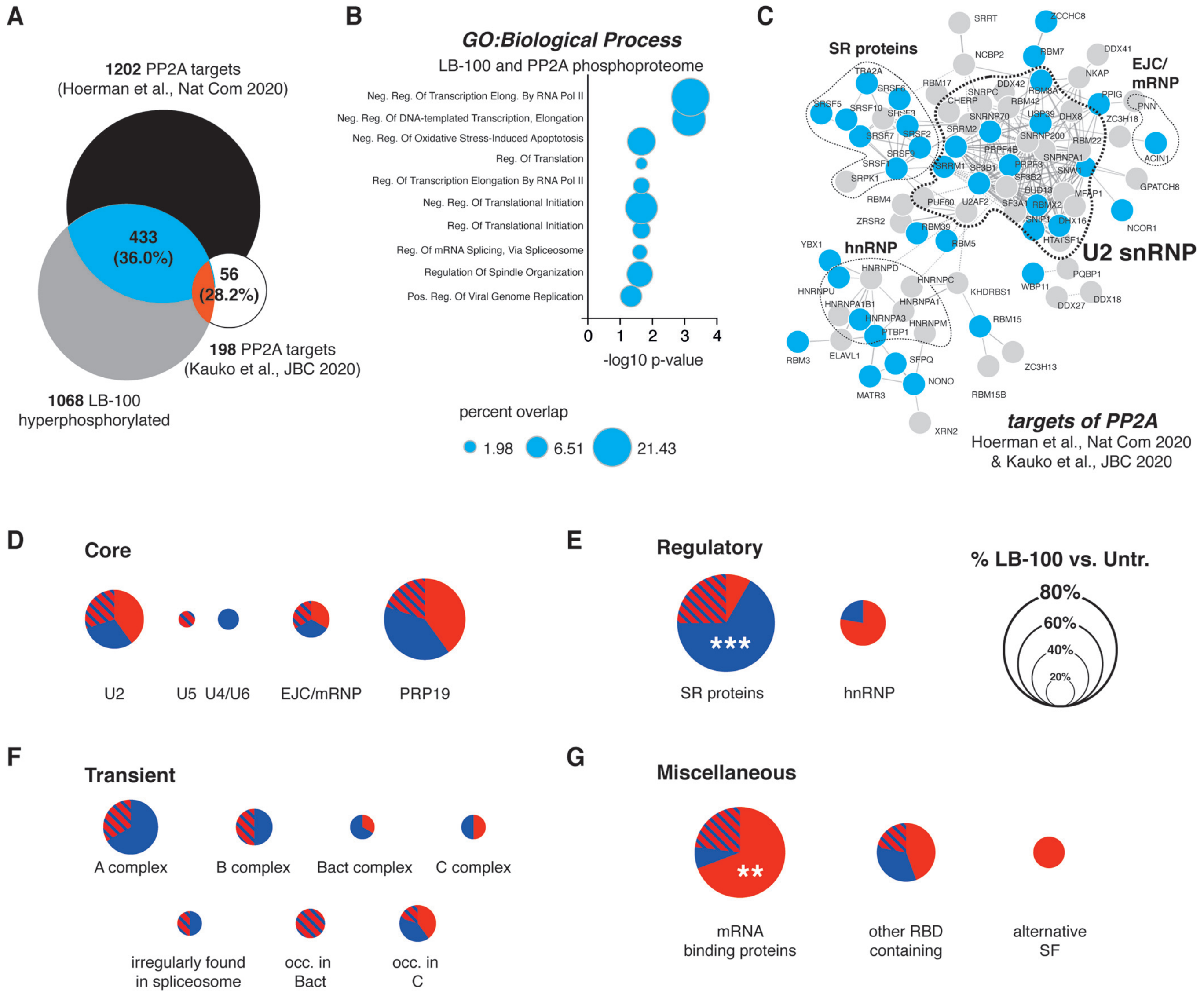

Figure EV1. Specific splicing nodes are differentially phosphorylated in response to LB-100 treatment in colorectal adenocarcinoma.

(**A**) Venn diagram shows an overlap between differentially hyperphosphorylated proteins in LB-100-treated SW-480 colon adenocarcinomas and previously reported targets of PP2A (Hoermann et al, 2020; Kauko et al, 2020). (**B**) The bar graph shows Gene Ontologies enriched for 34 differentially phosphorylated proteins from LB-100-treated SW-480 human colorectal carcinoma that are previously reported PP2A targets (Hoermann et al, 2020; Kauko et al, 2020). *p* values were calculated with Fisher's exact test. (**C**) Interaction maps of spliceosome components hyper- and hypophosphorylated in response to 12 h of LB-100 stimulation in SW-480 cells (gray) overlaid with previously reported targets of PP2A (Hoermann et al, 2020; Kauko et al, 2020) (blue). Connecting lines show interactions from STRINGdb. (**D–G**) Pie charts show the distribution of proteins with significantly changed (FDR <0.05 and $|Log_2Fold\ Change| > 0.58$) phosphorylation status within different functional groups of splicing factors. RBD RNA binding domain, SF splicing factor. Data information: In (**D–G**), statistical significance was calculated with hypergeometric test vs. background, $^{***}p < 0.001$; $^{**}p < 0.01$.

 © The Author(s)

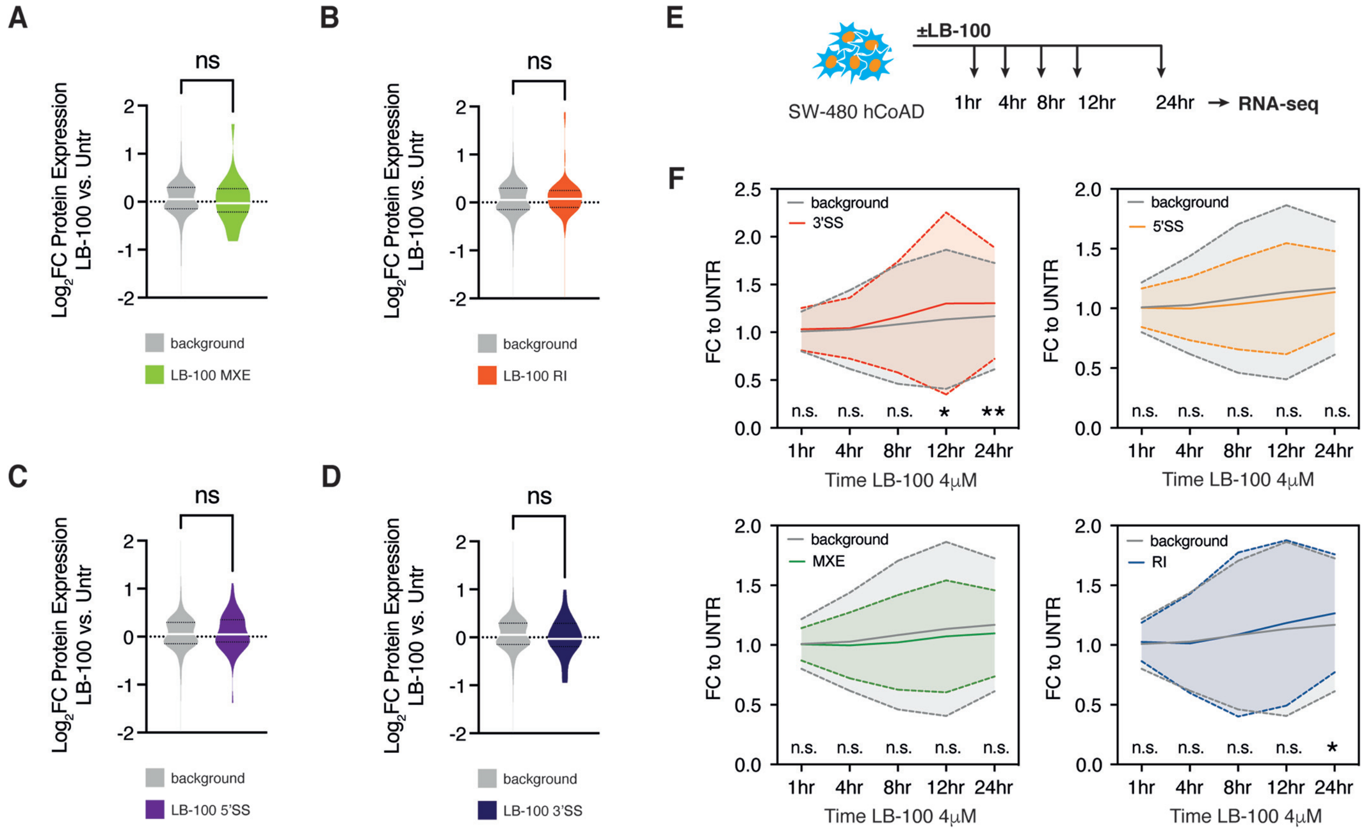

Figure EV2. PP2A inhibition-dependent alternative splicing affects protein but not transcript levels of alternatively spliced mRNAs.

(**A–D**) Violin plots show no effect on levels of proteins resulting from alternatively spliced transcripts in MXE (**A**), RI (**B**), alt5'ss (**C**), and alt3'ss (**D**) in 4 μM LB-100-treated SW-480 cells compared to control in $n = 4$ biological replicates. (**E**) Schematic illustrates an experimental approach to determine time-resolved transcriptomic changes of alternatively spliced mRNAs in response to 4 μM LB-100 stimulation. (**F**) Graphs show time-resolved changes in alternatively spliced transcripts and mRNAs from RI, MXE, alt5'ss, and alt3'ss groups in response to LB-100 stimulation. Data information: In (**F**), statistical significance was calculated with one-way ANOVA, $^{**}p < 0.01$; $^{*}p < 0.05$; n.s. not significant. Source data are available online for this figure.

© The Author(s)

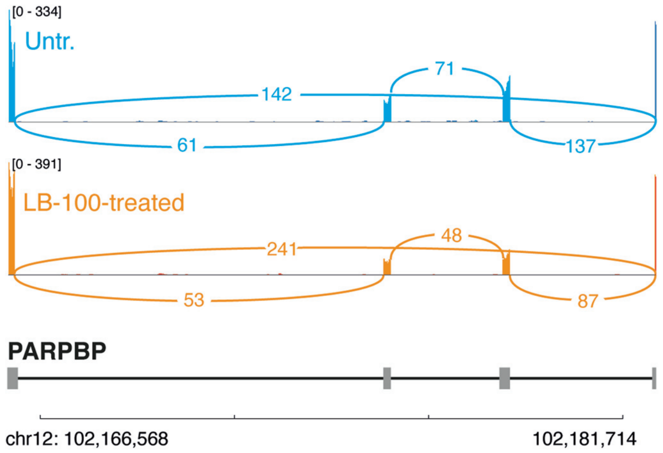

Figure EV3. Alternative splicing events in response to LB-100 impact the fate of DNA damage-response regulators.

An alternative splicing event within PARPBP gene and representative Sashimi plots showing differential splicing in untreated and LB-100-treated SW-480 cells.

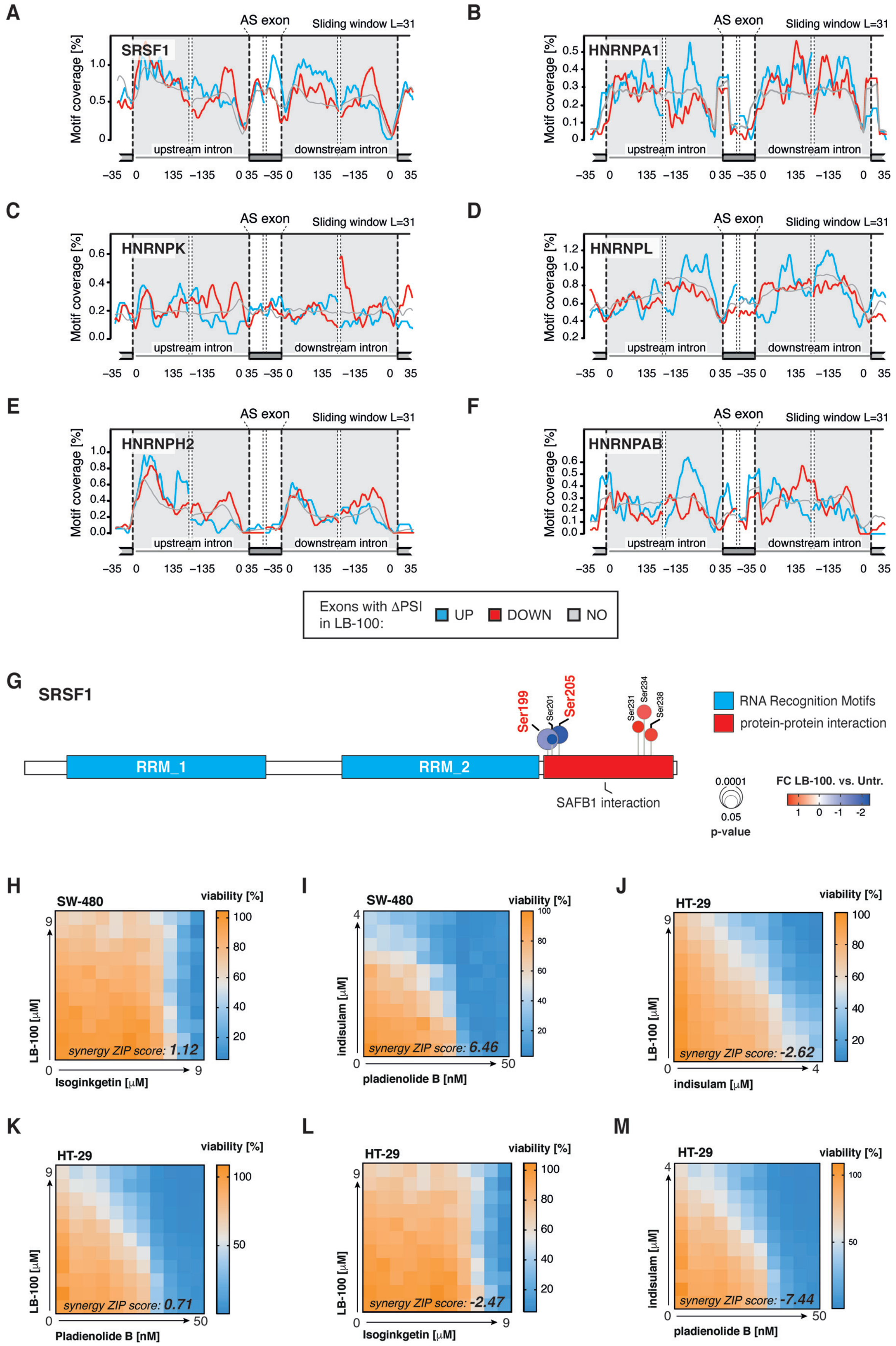
A
SRSF1
AS exon
Sliding window L=31
Motif coverage [%]
upstream intron
downstream intron
B
HNRNPA1
C
HNRNPK
D
HNRNPL
E
HNRNPH2
F
HNRNPAB
Exons with ΔPSI in LB-100:
UP
DOWN
NO
G
SRSF1
RRM_1
RRM_2
Ser199
Ser201
Ser205
Ser231
Ser234
Ser238
SAFB1 interaction
RNA Recognition Motifs
protein-protein interaction
p-value
FC LB-100. vs. Untr.
H
SW-480
viability [%]
synergy ZIP score: 1.12
LB-100 [μM]
Isoginkgetin [μM]
I
SW-480
synergy ZIP score: 6.46
indisulam [μM]
pladienolide B [nM]
J
HT-29
synergy ZIP score: -2.62
LB-100 [μM]
indisulam [μM]
K
HT-29
synergy ZIP score: 0.71
LB-100 [μM]
Pladienolide B [nM]
L
HT-29
synergy ZIP score: -2.47
LB-100 [μM]
Isoginkgetin [μM]
M
HT-29
synergy ZIP score: -7.44
indisulam [μM]
pladienolide B [nM]

Figure EV4. Impact of LB-100-driven phosphorylation changes on the function of spliceosome components.

(**A–F**) Motif RNA map shows SRSF1 (**A**), HNRNPA1 (**B**), HNRNPK (**C**), HNRNPL (**D**), HNRNPH2 (**E**), and HNRNPAB (**F**) proteins binding in the proximity of excluded (DOWN), included (UP), or not affected (NO) exons in LB-100-treated as compared to untreated SW-480 colorectal adenocarcinoma cells. (**G**) Location of mis-phosphorylated sites across SRSF1 protein. Functionally relevant protein regions according to UniProt are shown. Significantly changing phosphosites in response to LB-100 are highlighted. (**H**,**I**) Synergy matrices show a lack of cooperativity between LB-100 and isoginkgetin (**H**) or indisulam and pladienolide B (**I**) in the SW-480 human adenocarcinoma cell line. Representative heat map for $n = 3$ biological replicates. (**J–M**) Synergy matrices show a lack of cooperativity between LB-100 and indisulam (**J**), pladienolide B (**K**), isoginkgetin (**L**), or indisulam and pladienolide B (**M**) in HT-29 human adenocarcinoma cell line. Representative heat map for $n = 3$ biological replicates. Source data are available online for this figure.

 © The Author(s)

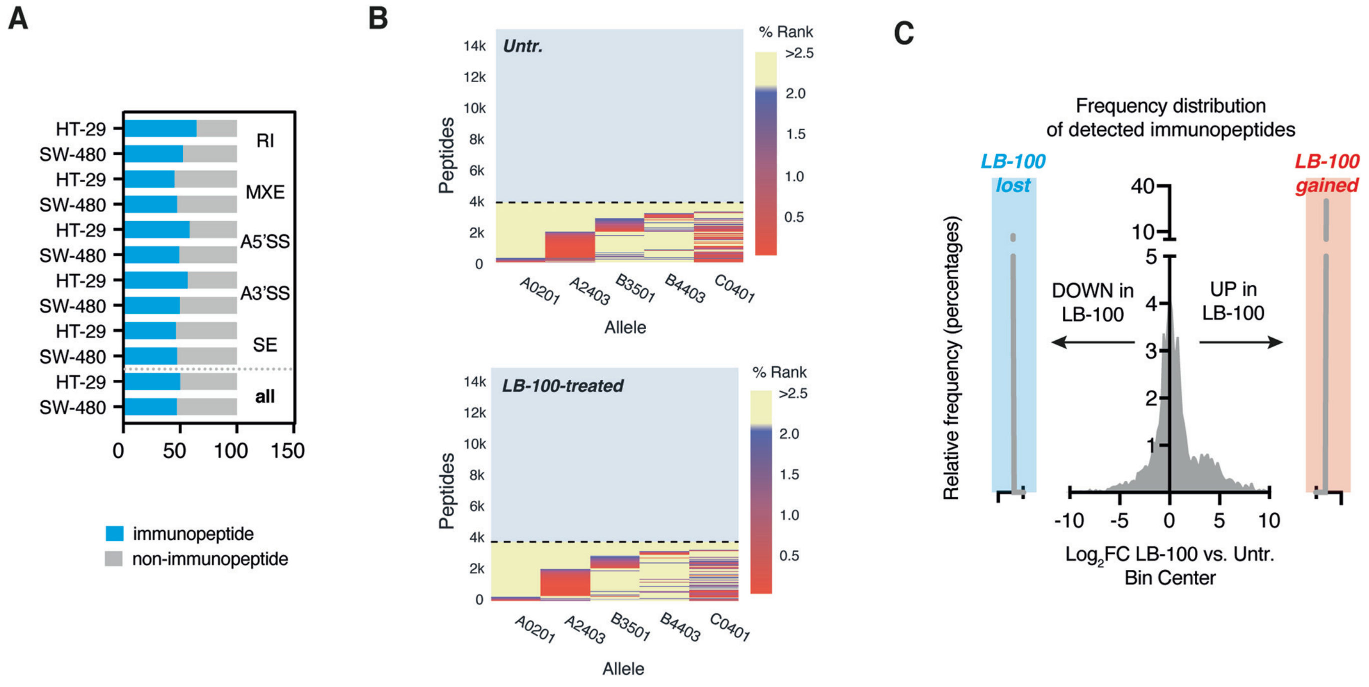

Figure EV5. LB-100 determines neoantigen formation presented within MHC-I.

(**A**) The bar graph shows an equal contribution of all the splicing events toward the potential formation of tumor neoantigens reported previously (Lu et al, 2021). (**B**) Heat maps show HLA haplotype representation in untreated controls (Untr.). and LB-100-stimulated HT-29 human colorectal carcinoma cells from $n = 3$ biological replicates. (**C**) The plot shows the cumulative distribution of $\log_2$ fold change in peptides presentation between LB-100-stimulated and untreated control (Untr.) HT-29 cells from $n = 3$ biological replicates. Highlighted in red and blue are peptides detected exclusively in LB-100 and control cells, respectively.

© The Author(s)
